# Can Pseudothrombocytopenia be recognised at first look?

**DOI:** 10.1097/MD.0000000000035395

**Published:** 2023-10-13

**Authors:** Seda Yilmaz, Mikail Dağ, Muhammet Cemal Kizilarslanoğlu, Abdulkadir Baştürk

**Affiliations:** a Department of Internal Medicine, Clinic of Hematology, Konya City Hospital, Konya, Turkey; b Department of Internal Medicine, Şanliurfa Training and Research Hospital, Şanliurfa, Turkey; c Department of Internal Medicine, Division of Geriatrics, University of Health Sciences, Konya City Hospital, Konya, Turkey.

**Keywords:** immune thrombocytopenia, leukaemia, platelet, pseudothrombocytopenia

## Abstract

Our aim was to determine the laboratory parameters that distinguish pseudothrombocytopenia from true thrombocytopenia. A total of 107 patients who were referred to the adult hematology outpatient clinic with thrombocytopenia and subsequently diagnosed with acute myeloid leukaemia, immune thrombocytopenia and pseudothrombocytopenia were included in our study. Hemogram parameters on admission, platelet value in the control hemogram and peripheral smear findings were recorded. Forty three (40.2%) males and 64 (59.8%) females, were included in our study. There were 25 patients in the leukaemia group, 39 in the immune thrombocytopenia group and 43 in the pseudothrombocytopenia group. Control platelet value and red cell distribution width/platelet ratio were found to be statistically significantly different between the 3 groups. Receiver operating characteristic analysis based on platelet values showed that platelet value ≤ 38,000/µL (86% sensitivity, 78.1% specificity, *P* < .001), difference between 2 consecutively measured platelet levels ≤ 11. 000/µL (79.1% sensitivity, 79.7% specificity, *P* < .001), red cell distribution width/platelet ratio ≥ 0.413 (90.7% sensitivity, 78.1% specificity, *P* < .001) were found to be in favor of true thrombocytopenia. In the differentiation of pseudothrombocytopenia and true thrombocytopenia, the difference between the hemogram parameters at the time of admission and the platelet count in the control blood count may be guiding. This result may reduce patient and physician anxiety and prevent patient referral.

## 1. Introduction

Thrombocytopenia is defined as a platelet count (PLT) < 150 × 10^3^/µL and can be divided into 100 to 150 × 10^3^/µL mild, 50 to 99 × 10^3^/µL moderate, <50 × 10^3^/µL severe.^[1]^ Thrombocytopenia may be associated with a condition that carries a risk of life-threatening bleeding or thrombosis.^[2–4]^ Therefore, the etiology of thrombocytopenia is important in the treatment management of thrombocytopenia.

Pseudothrombocytopenia (PTCP) was first described in a patient with non-Hodgkin lymphoma and has been recently reported in patients with coronavirus-19 infection.^[5,6]^ This can lead to misdiagnosis of thrombocytopenia, overtreatment and even invasive, unnecessary tests.

Primary immune thrombocytopenia (ITP) is isolated thrombocytopenia and is diagnosed by exclusion of other causes and there is no gold standard test.^[7]^ Most patients are asymptomatic, but 5-6% of patients may be admitted with severe hemorrhage.^[8]^ ITP has also been misdiagnosed in cases of thrombocytopenia due to secondary causes and this rate has been reported as 20% in various studies.^[9–11]^

Thrombocytopenia may also be seen in acute myeloid leukaemia (AML) and life-threatening hemorrhages may be observed.^[2]^

It is important to determine whether the thrombocytopenia detected on admission is true thrombocytopenia or not; if true thrombocytopenia, it is important to differentiate between the many possible causes and to determine the risks of bleeding, thrombosis and other potential complications and to refer to the relevant clinic if necessary. For these reasons, we planned this study to answer the questions “can we differentiate PTCP from other true thrombocytopenia during the first look at the complete blood count?,” “which parameters are important in the differentiation of PTCP, AML or ITP?,” “is there a platelet basal threshold value in the differential diagnosis?,” “is the difference in platelet count between 2 blood counts significant?”.

## 2. Materials and methods

### 2.1. Participants and procedure

A total of 107 adult patients diagnosed with AML, ITP and PTCP who were referred to the hematology outpatient clinic with thrombocytopenia between September 2021 to September 2022 were included in our study. Patients who were referred for thrombocytopenia but diagnosed other than these diagnoses were not included in the study. Hemogram parameters on admission, platelet value in the control hemogram and peripheral smear findings were recorded. Patients were divided into 2 groups as PTCP and true thrombocytopenia and analyzed. Then PTCP and ITP were compared and PTCP and AML groups were compared.

The ethical approval for the study was obtained from the ethics committee of Medical Faculty (decision date: September 21, 2022 and no. 2022/027).

### 2.2. Data analysis

The data were entered into the computer programme SPSS 26.0. The suitability of the data for normal distribution was evaluated according to Skewness-Kurtosis, Kolmogorov-Smirnov test, coefficient of variation and histogram data. Non-normally distributed data were shown as median and minimum-maximum, and categorical data were shown as number and percentage.

Comparison of numerical data in 2 groups was performed by Mann–Whitney *U* test. Kruskal Wallis test was used to compare the medians in more than 2 groups. Statistical analyses were performed with Bonferroni corrected post hoc Mann–Whitney *U* test. Comparison of categorical variables was performed with Chi-square or Fisher Exact tests. A *P* value < .05 was accepted for the significance of all data obtained after data analysis. Platelet-related data were recorded in the MedCalc programme between the groups. Receiver operating characteristic (ROC) analysis was performed.

## 3. Results

### 3.1. Comparison between the AML, ITP, and PTCP groups

A total of 107 patients, 43 (40.2%) males and 64 (59.8%) females, were included in our study. When divided into age groups, 31 (29.0%) of the participants were aged between 18 to 35 years, 28 (26.2%) were aged between 36 to 50 years, 27 (25.2%) were aged between 51 to 65 years and 21 (19.6%) were over 66 years. Among the participants, 85 (79.4%) were in the young group (<65 years) and 22 (20.6%) were in the elderly group (65 years and older). Patients were divided into groups according to the diagnoses of AML, ITP, and PTCP. No significant difference was found between the groups divided according to diagnosis and the groups divided according to gender and age. Nonparametric group comparisons are shown in Table 1.

**Table 1:** Nonparametric group comparisons.

		Total (Percentage)	AML	ITP	PTCP	*P* value
Gender	Male	43 (%40.2)	13 (%52.0)	16 (%41 0)	14 (%32.6)	.286
	Female	64 (%59.8)	12 (%48.0)	23 (%59.0)	29 (%64.4)	
Age group	18–35	31 (%29.0)	4 (%16.0)	11 (%28.2)	16 (%37.2)	.142
	36–50	28 (%26.2)	4 (%16.0)	13 (%33.3)	11 (%25.6)	
	51–65	27 (%25.2)	10 (%40.0)	10 (%25.6)	7 (%16.3)	
	>65	21 (%19.6)	7 (%28.0)	5 (%12.8)	9 (%20.9)	
Young-elder	Young (<65)	85 (%79.4)	17 (%68.0)	34 (%79.1)	34 (%79.1)	.179
	Elder (≥65)	22 (%20.6)	8 (%32.0)	5 (%20.9)	9 (%20.9)	

AML = acute myeloid leukaemia, ITP = primary immune thrombocytopenia, PTCP = pseudothrombocytopenia.

The median age of the individuals included in the study was 48 (19–88). No significant difference was found between the diagnostic groups according to age (*P*: .057). In the comparison of white blood cell (WBC) levels, although the lowest level was found in the AML group with 4.01 (0.73–174.53), no significant difference was found between the groups. Compared to the other groups, in the AML group; red blood cell (RBC), hemoglobin, hematocrit, neutrophil (NE), eosinophil, basophil, NE percentage, basophil percentage, lymphocyte (LYM) percentage, eosinophil percentage, platelet distribution width values were lower (*P* < .001; *P* < .001; *P* < .001; *P* < .001; *P* < .001; *P* < .001; p:0.001; *P* < .001; *P* < .001; *P* < .001; *P*: .005, respectively) in AML group, mean corpuscular volume, mean corpuscular hemoglobin concentration, monocyte count, percentage of monocyte count, red cell distribution width (RDW) -PLT ratio values were higher and this was statistically significant (*P* < .001; *P* < .001; *P* < .001; p:0.024; *P* < .001; *P* < .001; *P* < .001; *P*: .003, respectively). When the statistically significant differences between the ITP group and the other groups were analyzed, PLT, platecrit (PCT), PLT/LYM ratio and PLT/WBC ratio were lower (*P* < .001 for all), while mean platelet volume (MPV), NE/PLT ratio and RBC/PLT ratio were higher (*P*: .004; *P* < .001; *P* < .001, respectively). Control PLT and RDW/PLT ratio were found to be statistically significantly different between the 3 groups (*P* < .001 for 2 parameters). The data obtained as a result of the comparison of parametric data are shown in Table 2.

**Table 2:** Comparison of parametric data.

	Median (min-max)	AML	ITP	PTCP	*P* value	AML/ITP	AML/PTCP	ITP/PTCP
Age (yr)	48 (19–88)	58 (20–81)^a^	45 (19–88)^a^	44 (23–83)^a^	.057	0.016	0.057	0.963
WBC (10^3^/µL)	7.19 (0,73–174.53)	4.01 (0.73–174.53)^a^	8.25 (2.26–20.35)^a^	6.58 (3.94–12.54)^a^	.048	0.056	0.094	0.072
RBC (106/µL)	4.45 (1.42–6.51)	2.62 (1.42–5.57)^a^	4.42 (1.69–6.51)^b^	4.84 (3.67–6.4)^b^	<.001	<0.001	<0.001	0.043
HGB (g/dL)	12.8 (4.8–17)	8.1 (4.8–14.5)^a^	12.7 (5.3–17)^b^	13.8 (10.1–16.8)^b^	<.001	<0.001	<0.001	0.015
HCT (%)	38.7 (14.9–53.4)	25.4 (14.9–44.3)^a^	38.3 (16.7–53.4)^b^	42.8 (31.5–52.5)^b^	<.001	<0.001	<0.001	0.001
MCV (fL)	88.6 (55.9–114)	98.2 (74.4–114)^a^	85 (55.9–98.8)^b^	88.6 (64.1–95.2)^b^	<.001	<0.001	<0.001	0.009
MCH (pg)	29.4 (15.8–39.5)	32.7 (24.5–39.5)^a^	28.8 (15.8–32)^b^	29.2 (18.8–32.3)^b^	<.001	<0.001	<0.001	0.231
MCHC (g/dL)	32.9 (27.9–36.7)	33.2 (27.9–36.7)^a^	33.2 (28.3–35.1)^a^	32.5 (28–35.5)^a^	.117	0.767	0.095	0.073
PLT (10^3^/µL)	36 (1–148)	42 (10–132)^a^	16 (1–56)^b^	94 (8–148)^a^	<.001	<0.001	0.004	<0.001
MPV (fL)	12.3 (8.7–15)	11.25 (8.7–13.2)^a^	13 (10.7–15)^b^	12.35 (9.3–14.4)^ab^	.004	0.002	0.048	0.022
PCT (%)	0.1 (0.01–0.18)	0.08 (0.01–0.14)^ab^	0.03 (0.02–0.1)^a^	0.13 (0.01–0.18)^b^	<.001	0.043	0.002	<0.001
NE (10^3^/µL)	3.77 (0.09–26.13)	1.36 (0.09–26.13)^a^	4.96 (1.65–15.18)^b^	3.7 (1.82–11.38)^b^	<.001	<0.001	<0.001	0.043
LY (10^3^/µL)	1.97 (0.4–17.39)	2.15 (0.4–17.39)^a^	1.97 (0.57–4.81)^a^	1.86 (0.77–6.02)^a^	.830	0.500	0.794	0.773
MO (10^3^/µL)	0.53 (0.04–144.89)	0.99 (0.07–144.89)^a^	0.58 (0.04–1.73)^b^	0.46 (0.15–0.76)^b^	.024	0.107	0.038	0.030
EO (10^3^/µL)	0.07 (0–2.01)	0.01 (0–0.24)^a^	0.08 (0–2.01)^b^	0.12 (0–0.53)^b^	<.001	<0.001	<0.001	0.167
BA (10^3^/µL)	0.02 (0–0.26)	0.01 (0–0.26)^a^	0.02 (0–0.14)^ab^	0.02 (0.01–0.06)^b^	.001	0.002	<0.001	0.615
NE%	57.1 (2.3–91.4)	21 (2.3–55.2)^a^	62 (44.6–91.4)^b^	58.7 (31.7–90.8)^b^	<.001	<0.001	<0.001	0.081
LY%	30.4 (3.9–80.4)	41.7 (3.9–80.4)^a^	25.6 (7–43.6)^b^	31 (7.7–60.9)^ab^	<.001	<0.001	0.010	0.041
MO%	7.8 (1.1–83)	21.6 (4.5–83)^a^	7.3 (1.1–12.4)^b^	6.9 (1.2–12.8)^b^	<.001	<0.001	<0.001	0.307
EO%	1 (0–23)	0 (0–2.6)^a^	0.9 (0–23)^b^	1.5 (0–6.3)^b^	<.001	0.001	<0.001	0.065
BA%	0.2 (0–1.2)	0 (0–0.4)^a^	0.3 (0–1)^b^	0.3 (0.1–1.2)^b^	<.001	<0.001	<0.001	0.183
PDW (fL)	15.2 (0–37.6)	12.2 (6.7–19.5)^a^	17.3 (0–37.6)^ab^	15.4 (0––21.7)^b^	.005	0.012	0.003	0.228
RDW_SD (fL)	43.7 (0.2–95.2)	60.2 (42.5–95.2)^a^	41.2 (0.2–57.1)^b^	42.9 (33.8–54.6)^b^	<.001	<0.001	<0.001	0.046
RDW_CV (%)	14.2 (11.7–57)	18.9 (13.3–28.9)^a^	13.7 (11.7–57)^b^	13.5 (11.9–19)^b^	<.001	<0.001	<0.001	0.400
Control PLT(10^3^/µL)	45 (2–377)	45 (8–139)^a^	18 (2–67)^b^	124 (19–377)^c^	<.001	<0.001	<0.001	<0.001
PLT difference (10^3^/µL)	9 (0–291)	5 (0–35)^a^	4 (0–44)^a^	26 (1–291)^b^	<.001	0.989	<0.001	<0.001
PLT/control PLT	0.94 (0.06–5.05)	1.05 (0.22–2.38)^a^	0.9 (0.1–2.33)^ab^	0.83 (0.06–5.05)^b^	.003	0.022	0.001	0.378
RDW/PLT	0.42 (0.083–14.1)	0.42 (0.13–1.56)^a^	0.99 (0.26–14.1)^b^	0.15 (0.08–1.63)^c^	<.001	<0.001	<0.001	<0.001
NE/PLT	0.077 (0.0015–5.95)	0.03 (0–0.93)^a^	0.44 (0.05–5.95)^b^	0.05 (0.01–0.82)^a^	<.001	<0.001	0.056	<0.001
PLT/LYM	19.3 (0.5–116.9)	19.66 (1.44–102.04)^a^	7.46 (0.51–35.09)^b^	51.49 (4.39–116.88)^a^	<.001	<0.001	0.005	<0.001
PLT/WBC	6.48 (0.11–64.1)	9.51 (0.15–64.1)^a^	1.83 (0.11–10.85)^b^	12.64 (0.97–31.68)^a^	<.001	<0.001	0.343	<0.001
RBC/PLT	0.1 (0.0126–5.2)	0.05 (0.01–0.25)^a^	0.28 (0.05–5.2)^b^	0.05 (0.03–0.61)^a^	<.001	<0.001	0.698	<0.001

AML = acute myeloid leukaemia, BA = basophil, EO = eosinophil, HGB = hemoglobin, HCT = hematocrit, ITP = primary immune thrombocytopenia, LYM = lymphocyte, MCHC = mean corpuscular hemoglobin concentration, MPV = mean platelet volume, PCT = platecrit, PLT = platelet, PTCP = pseudothrombocytopenia, PDW = platelet distribution width, RBC = red blood cell, RDW = red cell distribution width, WBC = white blood cell.

### 3.2. Comparison between the true thrombocytopenia and PTCP groups

When the AML and ITP groups were combined and compared with the PTCP group on the basis of platelet values, it was found that PLT, PCT, control PLT, PLT difference, PLT/LYM ratio and PLT/WBC ratio were statistically significantly lower in the group with true thrombocytopenia (*P* < .001 for all), while PLT/control PLT ratio, RDW/PLT ratio, neutrophil (NEU)/PLT ratio and RBC/PLT ratio were higher (*P*: .019; *P* < .001; *P* < .001; *P* < .001, respectively). The data obtained from the comparison between PTCP and true thrombocytopenia groups are shown in Table 3.

**Table 3:** Comparison of parametric data between true thrombocytopenia and PTCP groups.

	True thrombocytopenia	PTCP	*P* value
PLT(10^3^/µL)	23.5 (1–132)	94 (8–148)	<.001
PCT (%)	0.04 (0.01–0.14)	0.13 (0.01–0.18)	<.001
PDW(fL)	13.5 (0–37.6)	15.4 (0–21.7)	.237
Control PLT(10^3^/µL)	25 (2–139)	124 (19–377)	<.001
PLT difference (10^3^/µL)	5 (0–44)	26 (1–291)	<.001
PLT/control PLT	1 (0.1–2.38)	0.83 (0.06–5.05)	.019
RDW/PLT	0.76 (0.13–14.1)	0.15 (0.08–1.63)	<.001
NEU/PLT	0.19 (0–5.95)	0.05 (0.01–0.82)	<.001
PLT/LYM	11.44 (0.51–102.04)	51.49 (4.39-116.88)	<.001
PLT/WBC	2.69 (0.11–64.1)	12.64 (0.97–31.68)	<.001
RBC/PLT	0.16 (0.01–5.2)	0.05 (0.03–0.61)	<.001

LYM = lymphocyte, NEU = neutrophil, PCT = platecrit, PLT = platelet, PTCP = pseudothrombocytopenia, PDW = platelet distribution width, RBC = red blood cell, RDW = red cell distribution width, WBC = white blood cell.

When AML and PTCP groups were compared, ROC analysis based on statistically significant platelet values revealed that the probability of PTCP was higher when PLT > 42.000/µL with a sensitivity of 83.7% and specificity of 52% (*P*: .001). In particular, the difference between 2 consecutively measured platelet levels > 11.000/µL was found to be in favor of PTCP with 79.1% sensitivity and 80% specificity (*P* < .001). ROC analysis results between AML and PTCP are shown in Table 4.

**Table 4:** AML and PTCP groups ROC analysis results.

	Cutoff	AUC	Sensitivity (%)	Specificity (%)	NPV	PPV	*P* value
PLT (10^3^/µL)	>42	0.712	83.7	52	65	75	.001
Control PLT (10^3^/µL)	>51	0.811	83.7	60	68.2	78.3	<.001
PLT difference (10^3^/µL)	>11	0.841	79.1	80	69	87.2	<.001
PLT/control PLT	≤0.89	0.754	62.8	92	59	93.1	<.001
RDW/PLT	≤0.156	0.786	53.5	92	53.5	92	<.001

AML = acute myeloid leukaemia, PLT = platelet, PTCP = pseudothrombocytopenia, RDW = red cell distribution width, ROC = receiver operating characteristic.

When ITP and PTCP groups were compared, ROC analysis based on statistically significant platelet values revealed that platelet counts of 37 or less were in favor of ITP (sensitivity: 86.05%, specificity: 97.4%, *P* < .001). A difference of more than 11 between 2 consecutive platelet counts was found to be more likely to be PTCP with 79.07% sensitivity and 79.49% specificity (*P* < .001). It was concluded that RDW/PLT ratio and RBC/PLT ratio had high sensitivity (88.37% and 86%, respectively) and specificity (97.37% and 92.5%, respectively) for PTCP (*P* < .001). ROC analysis results between ITP and PTCP are shown in Table 5.

**Table 5:** ITP and PTCP groups ROC analysis results.

	Cutoff	AUC	Sensitivity	Specificity	NPV	PPV	*P* value
PLT (10^3^/µL)	>37	0.937	86.05	97.44	86.4	97.4	<.001
PCT (%)	>0.1	0.9	68.4	100	52	100	<.001
Control PLT(10^3^/µL)	>49	0.967	88.37	97.44	88.4	97.4	<.001
PLT difference (10^3^/µL)	>11	0.832	79.07	79.49	77.5	81	<.001
RDW/PLT	≤0.389	0.942	88.37	97.37	88.4	97.4	<.001
NEU/PLT	≤0.125	0.932	90.7	82.05	88.9	84.8	<.001
PLT/LYM	>35.088	0.909	69.77	100	75	100	<.001
PLT/WBC	>6157	0.936	83.72	92.31	83.7	92.3	<.001
RBC/PLT	≤0.121	0.93	86	92.3	85.7	92.5	<.001

ITP = primary immune thrombocytopenia, LYM = lymphocyte, NEU = neutrophil, PCT = platecrit PLT = platelet, PTCP = pseudothrombocytopenia, RBC = red blood cell, RDW = red cell distribution width, ROC = receiver operating characteristic, WBC = white blood cell.

When true thrombocytopenia and PTCP groups were compared, the lowest platelet count measured was 38 or lower (86% sensitivity, 78.1% specificity, *P* < .001), and difference of 11 or less between 2 measured platelet levels (79.1% sensitivity, 79.7% specificity, *P* < .001), and an RDW/PLT ratio > 0.413 (90.7% sensitivity, 78.1% specificity, *P* < .001) were found to be in favor of true thrombocytopenia. The results of the ROC analysis between true thrombocytopenia and PTCP are shown in Table 6.

**Table 6:** ROC analysis results for true thrombocytopenia and PTCP groups.

	Cutoff	AUC	Sensitivity	Specificity	NPV	PPV	*P* value
PLT (10^3^/µL)	>38	0.849	86	78.1	89.3	72.5	<.001
PLT difference (10^3^/µL)	>11	0.836	79.1	79.7	85	72.3	<.001
NEU/PLT	≤0.125	0.709	90.7	57.8	90.2	59.1	<.001
PLT/LYM	>35.088	0.83	69.8	89.1	81.4	81.1	<.001
PLT/WBC	>6484	0.793	83.7	73.4	87	67.9	<.001
RBC/PLT	≤0.13	0.751	88.4	62.5	88.9	61.3	<.001
RDW/PLT	≤0.413	0.881	90.7	78.1	92.6	73.6	<.001

LYM = lymphocyte, NEU = neutrophil, PLT = platelet, PTCP = pseudothrombocytopenia, RBC = red blood cell, RDW = red cell distribution width, ROC = receiver operating characteristic, WBC = white blood cell.

## 4. Discussion

Although various ratios such as PLT/LYM, PLT/WBC, RDW/PLT, NEU/PLT, platelet ratio in 2 counts, RBC/PLT are meaningful in differentiating PTCP from true thrombocytopenia, it is important to perform a second control blood count and to pay attention to the difference between the platelet threshold value and the platelet values between the 2 counts in order to be usable and memorable in daily practice.

The prevalence of PTCP has been reported at different rates in retrospective studies, with a prevalence of 0.03 to 0.27% and up to 15% in patients presenting with isolated thrombocytopenia.^[12–23]^ This significant prevalence rate makes differentiation more important. Although the prevalence of PTCP does not appear to be significantly affected by age or gender (22), a retrospective study of 190,940 individuals showed that it was significantly higher in men aged 50 years and older.^[13]^ In our study, 32.6% of the PTCP group were male and 64.4% were female. Median age was 44 (23–83) years. When all 3 groups were divided as 18 to 35, 36 to 50, 51 to 65, >65 years and < 65, ≥65 years, no difference in gender distribution was observed.

Hemogram parameters may help to differentiate PTCP. The mean PLT value was 23.5 (1–132) 103/µL in the true thrombocytopenia group and 94 (8–148) 103/µL in the PTCP group. MPV is an indicator of platelet production and function. In some studies, elevated MPV was evaluated in favor of ITP.^[24,25]^ In another study, higher PCT and MPV levels in the PTCP group compared to the ITP group were evaluated in favor of PTCP. However, these parameters were not found to be significant in logistic regression analysis.^[26]^ In our study, PCT was lower and MPV was higher in the ITP group compared to the PTCP and AML groups. This result explains the etiology of thrombocytopenia and is consistent with the literature. Control PLT and RDW/PLT were statistically different between the 3 groups (*P* < .001 for 2 parameters). In our study, PLT, PCT, control PLT, PLT difference, PLT/LYM ratio and PLT/WBC ratio were statistically significantly lower in the group with true thrombocytopenia including ITP and AML patients (*P* < .001 for all), while PLT/control PLT ratio, RDW/PLT ratio, NEU/PLT ratio and RBC/PLT ratio were higher. When true thrombocytopenia and PTCP groups were compared, the lowest platelet count measured was 38 or lower (86% sensitivity, 78.1% specificity, *P* < .001), and a difference of 11 or less between 2 measured platelet levels (79.1% sensitivity, 79.7% specificity, *P* < .001), and RDW/PLT ratio >0.413 (90.7% sensitivity, 78.1% specificity, *P* < .001) were found to be in favor of true thrombocytopenia. In the literature, immature platelet fraction and reticulated platelets have been evaluated in PTCP, but no other parameters were observed.^[27,28]^ Therefore, we believe that evaluation using hemogram parameters will be useful in daily practice.

## 5. Conclusion

In our study, a platelet value of > 42.000/µL and a platelet count difference of > 11.000/µL between 2 counts were found in favor of PTCP. When thrombocytopenia cannot be evaluated with peripheral smear, especially control blood count should be performed; basal platelet value and high fluctuation between platelet values may be in favor of PTCP.

## Acknowledgments

I would like to thank the authors for their contributions

## Author contributions

**Data curation:** Seda Yilmaz, Mikail Dağ.

**Formal analysis:** Mikail Dağ, Muhammed Cemal Kizilarslanoğlu.

**Investigation:** Seda Yilmaz.

**Methodology:** Seda Yilmaz, Mikail Dağ, Muhammed Cemal Kizilarslanoğlu.

**Project administration:** Seda Yilmaz, Abdulkadir Baştürk.

**Resources:** Seda Yilmaz.

**Software:** Seda Yilmaz.

**Supervision:** Seda Yilmaz, Abdulkadir Baştürk.

**Validation:** Seda Yilmaz.

**Visualization:** Seda Yilmaz.

**Writing – original draft:** Seda Yilmaz.

**Writing – review & editing:** Seda Yilmaz.
